# Biostimulation as a Means for Optimizing Fruit Phytochemical Content and Functional Quality of Tomato Landraces of the San Marzano Area

**DOI:** 10.3390/foods10050926

**Published:** 2021-04-23

**Authors:** Youssef Rouphael, Giandomenico Corrado, Giuseppe Colla, Stefania De Pascale, Emilia Dell’Aversana, Luisa Ida D’Amelia, Giovanna Marta Fusco, Petronia Carillo

**Affiliations:** 1 Department of Agricultural Sciences, University of Naples Federico II, Via Università 100, 80055 Portici, Italy; youssef.rouphael@unina.it (Y.R.); depascal@unina.it (S.D.P.); 2 Department of Agriculture and Forest Sciences, University of Tuscia, Via San Camillo de Lellis, 01100 Viterbo, Italy; giucolla@unitus.it; 3 Department of Environmental, Biological and Pharmaceutical Sciences and Technologies, University of Campania “Luigi Vanvitelli”, Via Vivaldi 43, 81100 Caserta, Italy; emilia.dellaversana@unicampania.it (E.D.); luisa.damelia@unicampania.it (L.I.D.); giovannamarta.fusco@unicampania.it (G.M.F.); petronia.carillo@unicampania.it (P.C.)

**Keywords:** *Solanum lycopersicum* L., genetic variability, quality, food composition, biostimulant, plant tropical extract

## Abstract

The effect of plant biostimulation on fruits of traditional tomato germplasm is largely unknown. We examined how a tropical plant-derived biostimulant impacts the nutritional, functional, and compositional characteristics of tomato fruits from four landraces, collected in the San Marzano (SM) tomato Protected Designation of Origin (PDO) region, by profiling primary and secondary metabolites. Biostimulation was not able to completely reshuffle the morpho-physiological and nutritional profile of the four landraces. Their distinct phytochemical profile indicated a genotype-specific tuning of the analyzed traits, which also included an improved yield and fruit quality. Biostimulation of SM1 and SM3 increased photosynthetic accumulation of carbohydrate reserves, improved mineral nutrient use efficiency and consequently, yield (+21% and 34%, respectively). Moreover, biostimulation augmented the nutraceutical properties of the SM2 landrace. Interestingly, the plant-derived product increased in all genotypes lycopene, but not polyphenol accumulation in fruits. Our results show the potential of biostimulatory applications towards optimizing the fruit quality of the acclaimed SM landraces, which is suitable to satisfy both the rising consumer demand for premium traditional tomatoes and the technological needs of the food industry.

## 1. Introduction

In the last few decades, the consumer appreciation of the importance of food quality has been steadily increasing, regardless of the limitations related to the public perception of a multi-dimensional attribute [1]. Recently, a growing awareness of the environmental impact of the food production (in terms, for instance, of pollution, greenhouse gas emission, soil depletion, biodiversity loss, and chemical pesticides) has led to the definition of sustainable food quality [2]. A more sustainable food production system implies the use of resources at a rate that can be tolerated and ultimately, fully replenished by our environment. Sustainable food quality should consequently cover various issues, including safety, affordability, and nutritional and functional values, while controlling the use of chemical fertilizers, herbicides, and pesticides by leveraging natural plant defenses and biodiversity. The transition to a more sustainable food quality requires the promotion of agri-food systems and consumer behaviors that do not only emphasize aesthetic, nutritional, and functional attributes [3]. It is also necessary to put emphasis on safeguarding plant genetic resources to reverse agro-biodiversity loss, and reducing dependence on synthetic chemicals to limit the environmental impact of agriculture [4]. Under these perspectives, crop landraces are gaining popularity because of their perceived distinctive features and gastronomic value, as well as their amenability to more sustainable production systems (e.g., organic farming and low-input agriculture) and short food supply chains [5].

Tomato (*Solanum lycopersicum* L.) is one of the most widely grown vegetables, globally consumed fresh and in a variety of processed products. This species was domesticated in the Americas and, after the Columbian exchange, Italy and Spain have been recognized as secondary centers of diversification [6]. In these countries, several tomatoes with fruit shapes and colors different from the domesticated forms have been documented since its introduction [6]. Locally adapted tomato landraces can have interesting traits such as resistance to stress and high-quality fruits [7,8,9]. Contemporary varieties often out-yield landraces. Consequently, strengthening the use of landraces towards sustainable fruit quality necessitates strategies and tools to guarantee provenance and authenticity (e.g., for premium brands) [10,11,12], as well as non-regulatory initiatives in order to overcome technical barriers and to highlight commercially valuable features [5]. A sustainable strategy for increasing yield in landraces could be the use of plant biostimulants (PB). PBs are substances or microorganisms, not classified as fertilizer or pesticide, that can increase resource efficiency, growth, yield, and abiotic stress resilience and tolerance when applied to plants [13]. PBs mainly act on plants by inducing direct and indirect multiple physiological effects, which are linked, to name a few, to an increased mobility and solubility of mineral nutrients in soil, changes in root system architecture, improved water use efficiency, and enhanced ion uptake, mobilization and use [14]. With respect to the composition and properties of plant food, PBs can increase the synthesis and accumulation of primary and secondary metabolites, including important categories of antioxidants, such as carotenoids, polyphenols, and ascorbic acid, thus ultimately improving the nutritional and nutraceutical quality of the edible products [15,16,17]. Moreover, biostimulants can be also used for biofortification, for instance, improving the mineral content of leaves and fruits as well as their functional profile [18,19,20]. Biostimulants comprise various categories such as organic substances (e.g., humic acids, protein hydrolysates, chitosan, vitamins), inorganic compounds (e.g., cobalt, silica, selenium), and plant growth promoting microorganisms and their extracts (e.g., fungi, algae, bacteria) [13]. Nonetheless, it should not be overlooked that innovative products for agriculture, especially those suitable also for organic farming, should have the added benefit of making use of raw material that is disposed as organic waste of plant origin (plant bio-waste), to foster sustainable agricultural growth with minimal problems of biological and chemical safety [4,21].

In this work we tested the ability of a plant-derived biostimulant to enhance the nutritional, functional, and compositional characteristics of tomato fruits. While previous studies focused on modern, often hybrid, high-yield varieties bred for intensive agriculture [17], our aim was to understand the effect of biostimulation on a traditional germplasm that is culturally and gastronomically linked to a specified region. We focused on a biostimulant extracted from the biowaste of a tropical plant, which can be used in organic farming. We employed four distinct, indeterminate landraces whose fruit shapes typify the range of variability present in the whole peeled tomatoes grown in the area designated to produce the Protected Designation of Origins (PDO) San Marzano berries [22,23]. The interaction between the plant-based biostimulant and the different landraces over the fruit quality was assessed considering yield, as well as the mineral content, starch, soluble sugars, amino acids, proteins, lycopene, anthocyanins, and polyphenols of the fruits.

## 2. Materials and Methods

### 2.1. Plant Material, Growth Conditions and Experimental Design

This work was carried out on four tomato (*Solanum lycopersicum* L.) landraces collected in the area designated to produce the “Pomodoro San Marzano dell’Agro Sarnese-nocerino DOP”, the Protected Denomination of Origin (PDO) EU label scheme for the original San Marzano tomato [23]. These landraces were therefore named SM1, SM2, SM3, and SM4. The experiment was carried out in the 2017 summer growing season, in a greenhouse covered with a 0.25 mm thick ethyl vinyl acetate sheet at the experimental pilot farm “Torre Lama” (Bellizzi, SA) of the Department of Agricultural Sciences. The main physical and chemical soil characteristics at the experimental farm were clay loam texture with the following proportion of sand, silt, and clay: 47%, 25%, and 28%. The soil electrical conductivity (EC) was 0.15 dS m^−1^ and the pH 7.8. Total nitrogen was 0.11%, and organic matter: 1.23% (*w*/*w*). The Olsen phosphorus and exchangeable potassium were 85 and 889 mg kg^−1^, respectively. The tomato seedlings were transplanted on 2 May at the three-true-leaf stage. Plants grew under natural light conditions. The mean air temperature and relative humidity inside the greenhouse were 27.6 °C and 59%, respectively. Fertilizer was applied though drip irrigation system consisting of irrigation tubes placed 5 cm apart from the tomato plants, with holes spaced 0.3 m from each other, and an irrigation flow of 2.4 L·h^−1^. The NS delivered through fertigation was made (in mM) of N-NO_3_^−^: 12.0; S: 1.5; P: 1.0; K: 6.0; Ca: 3.5; Mg: 2.3; N-NH_4_^+^: 1.0. Micronutrients in the NS were (in μM): Fe: 20; Mn: 9; Cu: 0.3; Zn: 1.6; B: 20; Mo: 0.3. The pH and electric conductivity of the NS were 6.4 and 1.9 dS·m^−1^ at 25 °C, respectively. Fertigation was performed once per day. Landraces were treated by foliar application with a NS containing the commercial biostimulant Auxym^®^ (Italpollina, Rivoli Veronese, Italy), using as control treatment a no-biostimulant NS. The plant extract (PE) Auxym^®^ is produced through water extraction and fermentation of tropical plant biomass and its composition is presented elsewhere [24]. Plants were sprayed uniformly (four treatments) with a NS containing 2 mL·L^−1^ of Auxym^®^ using (PE treatment) or the NS (control treatment) using a 25-L tank weed sprayer, starting from the 35th day after the transplant (DAT). The experiment was carried out through a completely randomized design, namely four landraces (L), two biostimulant treatments (B), three replicates (R), resulting in 24 experimental units (4L × 2B × 3R).

### 2.2. Yield and Morphological Analysis

Considering the indeterminate growth pattern of the tomato landraces, fruit were continuously harvested starting 60 DAT (July 1) and continued until the end of the experiment (August 1; 90 DAT). During the harvest period, the marketable yield consisting of fully ripened fruits (mature red stage) was calculated on five plants located at the central part of each experimental unit. The fruits of the third truss were analyzed for quality parameters. Ten fresh fruits of each experimental unit were used for the biometric measurements (i.e., shape index, juice pH, and mineral content). The shape index of fruits was determined as a ratio of the maximum height length to maximum width, relative to the longitudinal section. The remaining subsamples were immediately frozen in liquid nitrogen and stored at −80 °C for further biochemical analyses.

### 2.3. Fruit Juice pH, Dry Matter Percentage and Ion Exchange Chromatography

Immediately after harvest, ten fresh tomato fruits of each experimental unit were homogenized in a blender (2 L; Waring HGB140, CA, USA) for one minute at low speed. The slurry was filtered through a two-layer cheesecloth and the juice pH was read with a digital pH meter (HI-9023; Hanna Instruments, Padua, Italy). The fruits’ dry matter percentage was also determined as a percentage of fresh mass following fruit desiccation to constant weight in a forced-air oven at 75 °C for 72 h, and weighed (Denver Instruments, Denver, CO, USA). Dried fruit tissues were ground in a Wiley Mill to pass through an 841 µm mesh and used for mineral analysis. For fruit mineral profile and citrate analysis, 250 mg of the dried material were suspended in 50 mL of ultrapure water (Milli-Q, Merck Millipore, Darmstadt, Germany), subjected to three freeze-thaw cycles in liquid nitrogen, centrifuged for 10 min at 6000 rpm (R-10 M, Remi Elektrotechnik Limited, India) and filtered through a 0.20 μm filter Whatman paper (Whatman International Ltd., Maidstone, UK). The clear supernatant was assayed by ion-exchange chromatography (ICS-3000, Dionex, Sunnyvale, CA, USA) as described [24]. Results of mineral composition were expressed in g kg^−1^ dw, except for nitrate that was converted to mg kg^−1^ fw based on each sample’s dw.

### 2.4. Starch and Soluble Carbohydrates Analysis

Starch and soluble sugars were determined as described by Carillo et al. [25] with some modifications. Fruits were frozen, finely ground, and 20 mg of powdered tissue were suspended in 250 µL of ethanol (98% *v/v* 5 mM Hepes/KOH; pH 7.0), incubated in a hot water bath (80 °C) for 20 min, and centrifuged at 14,000× *g* for 10 min at 4 °C. The clear supernatant was stored on ice. The pellet was submitted to two further extractions, first with 250 µL of 80% ethanol (*v*/*v*, 5 mM Hepes/KOH; pH 7.0), and then with 150 µL of 50% ethanol (*v*/*v*, 5 mM Hepes/KOH; pH 7.0), incubated and centrifuged as above. The supernatants of the three consecutive extractions were pooled and stored at −20 °C. The remaining pellet of the three ethanol extractions was used for starch determination. After the addition of 250 µL of 0.1 M KOH, samples were left at 90 °C for 2 h and then placed on ice. After cooling, the sample pH was brought to 4.5 by adding 75 µL of 1 M glacial acetic acid. An aliquot (100 µL) of acidified samples was added to 100 µL of 50 mM sodium acetate (pH 4.8) with 0.2 units α-amylase and 2 units amyloglucosidase and incubated at 37 °C for 18 h for starch hydrolysis. After centrifugation (14,000 rpm for 10 min at 4 °C), the soluble carbohydrates (fructose, glucose, sucrose) were analysed as previously detailed [26].

### 2.5. Antioxidant Metabolites Analysis

For anthocyanin quantification, frozen samples were finely powdered, and 40 mg were suspended in 200 µL of 40% (*v/v*) ethyl alcohol, thoroughly mixed, and incubated on ice for 20 min. After cold centrifugation (14,000 rpm, 10 min), the pellet was immediately extracted again using the same procedure. The two supernatants were then joined. Duplicate aliquots (150 µL) were dispensed in a microplate. Then, 75 µL of 25 mM KCl (pH 1.0) or 75 µL of 400 mM sodium acetate (pH 4.5) were added. A 150 µL of a 40% (*w*/*v*) ethanol solution was used as no-sample blank. The absorbance was read at 520 and 700 nm using a microplate-reading spectrophotometer (FLX-Xenius, SAFAS, Monaco, Germany). Quantification was performed as already reported [27]. Total anthocyanin content was expressed as mg cyanidin-3-glucoside equivalents per gram of fresh weight (mg C3G eq·g^−1^ FW).

Lycopene was evaluated using a previously published procedure with some modifications [28]. Powdered samples (20 mg) were suspended in 380 µL of solution of hexane, acetone, and methanol in a volume ratio of 2:1:1, containing also 0.05% (*w*/*v*) butylated hydroxytoluene. A no-plant extract was used as blank. The suspension was mixed on an orbital shaker for 30 min, centrifuged at 4 °C for 10 min at 14,000 rpm, and 100 µL of the orange organic phase were mixed with 1.4 mL hexane in a clean tube. The absorbance at 472 nm was measured as described above. Lycopene concentration was extrapolated with a calibration curve built with pure lycopene within the standard range (i.e., linear portion of the calibration curve) of 0.5–3 mg·L^−1^. Lycopene quantity was converted in mg·g^−1^ FW.

Polyphenols were determined as reported with some modifications [29]. Powdered samples (20 mg) were suspended in 800 μL of 60% methanol. The suspension was shaken at 800 rpm in a vortex mixer for 15 min and then centrifuged for 5 min at 8000× *g*. Aliquots (100 μL) of the clear supernatant or the same volume of 60% methanol (blank) were added to 50 μL of the Folin–Ciocalteu reagent diluted with distilled water (1:1 *v*/*v*). Samples were shaken for 6 min at 800 rpm, and then 650 μL of 3% (*w*/*v*) sodium carbonate were added. After an incubation of one hour and a half at room temperature, sample absorbance was spectrophotometrically read at 760 nm as described above. Polyphenols were quantified using a standard curve of gallic acid (GAE) in the 25–125 mg·L^−1^ range and expressed as mg GAE equivalents per gram of fresh weight (mg GAE·g^−1^ FW).

### 2.6. Quantification of Soluble Proteins and Free Amino Acids

The extraction of the soluble proteins was carried out starting from 20 mg of frozen tissue incubated at 4 °C for 24 h in 500 µL of Tris-HCl 200 mM (pH 7.5) containing 500 mM MgCl_2_. Samples were then centrifuged at 16,000× *g* for 10 min at 4 °C. For each biological replicate, triplicate aliquots (20 µL) of the clear supernatant, each with 180 µL of paper-filtered diluted (1:5 *v*/*v*) Protein Assay Dye Reagent Concentrate (Bio-Rad, Milan, Italy), were transferred to a 96–Well Flat–Bottom microtiter plate and thoroughly mixed. Protein standards (20 µL of 15, 37.5, 75, 112.5 and 150 mg·L^−1^, corresponding to 0.3, 0.75, 1.5, 2.25 and 3 µg of lyophilised BSA) were diluted in 200 mM Tris-HCl containing 500 mM MgCl_2_ as the samples. After incubation for at least 5 min at room temperature, absorbance was measured at 595 nm using a multi-detection microplate reader (Synergy HT, Biotek, Germany). Quantities were estimated using a blank corrected standard curve built with BSA and were expressed in mg·g^−1^ FW.

Amino acids were extracted essentially as described [30]. Samples (40 mg of powdered fruit tissue) were mixed with 1 mL of a 40% ethanol solution and left overnight at 4 °C. After cold-centrifuged at 14,000 rpm for 10 min. Primary AAs were analysed with a Nexera X2 Ultra High-Performance Liquid Chromatograph (Shimadzu, Milan, Italy), after an automated in needle three min pre-column derivatization of 20 μL of clear supernatant with 40 μL of derivatization solution [25]. Derivatized samples were injected onto a ZORBAX Eclipse Plus column (C18, 95 Å, 5 µm, 4.6 × 250 mm; Agilent Technologies, Milan, Italy) and eluted with a discontinuous gradient at 25 °C, with a 1 mL·min^−1^ flow rate. The detection of the amino acids (OPA-derivatized) was carried out using an excitation wavelength of 330 nm and reading emission at 450 nm. Peaks were assessed and quantified by comparing their relative retention time (RTT) and relative peak area (RPA) with that of injected reference standards [25]. Proline was quantified using the extract employed for the amino acids determination, employing an acid ninhydrin method according to a procedure previously described [31]. The amino acids were expressed as µmol·g^−1^ FW.

### 2.7. Net Economic Benefits: Partial Budget Analysis

A partial budget analysis was carried out essentially as described [18,32]. Briefly, a tomato selling price of 500 € t^−1^ at shipping point was used to calculate the added gross return of biostimulant-treated tomato production in comparison with untreated-tomato production. Moreover, the added variable costs (biostimulant product, foliar spraying, and fruit harvest of additional yield) were determined considering (i) a biostimulant selling price of 24 € L^−1^, (ii) a cost of single foliar spraying of 100 € ha^−1^, and (iii) hand-harvesting cost of 200 € t^−1^ [18]. The added net return was calculated as the difference between added gross return and added variable costs.

### 2.8. Statistical Analysis

A two-way Analysis of Variance (ANOVA) was carried out to examine the influence of the biostimulant treatment (B), the landrace (L), and their interaction (L × B). All data are presented as the mean ± Standard Error (SE). In the absence of significant L × B, mean separation was performed by Duncan’s Multiple Range Test (*p* < 0.05) for L and by Student’s *t*-test (*p* < 0.05) for B. For variables that were subject to significant L × B interaction, one-way ANOVA was performed according to Duncan’s multiple range test (*p* < 0.05). Calculations were performed using the SPSS 20 software (IBM, Akron, NY, USA). The Principal Component Analysis (PCA) was performed using the Minitab 18 statistical software (Minitab LLC, State College, PA, USA) [33].

## 3. Results and Discussion

### 3.1. Effects on Yield, Ecomomic Profitability and on Chemical and Physical Fruit Characteristics of the Genotype, Biostimulation and Their Interaction

**Figure 1:**
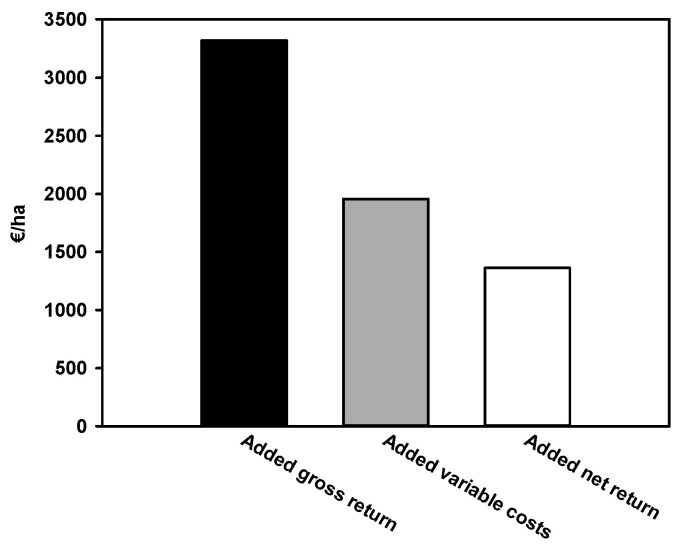
Added returns resulting from biostimulant applications on San Marzano tomato compared to untreated control.

Seeds of tomato landraces were collected from small farms in the area designated to produce the San Marzano PDO, and then multiplied and selected for uniformity at the Department of Agricultural Sciences, University of Naples Federico II. The four landraces under investigation were chosen as each had a distinctive fruit phenotype (Supplementary Table S1), thus representing a suitable panel of fruit diversity of the local landraces. The effect of the biostimulant over the tomato yield and its chemical and physical characteristics is reported in Table 1. Significant differences due to the factor “Landrace” (L) were present for the total yield and all the other parameters, except for the dry matter of the fruit, a parameter important mainly to produce tomato concentrated paste and puree [34]. Although trait variation was limited, the observed differences further indicated that, in addition to different fruit shapes, the four landraces have distinctive features. For the measured parameters, a significant interaction between the genotype and plant extract application (B) was not observed (Table 1). The main effect of the biostimulant treatment was more complex. Overall, the plant extract had a small positive effect on yield, but factors’ interaction had a main role, because yield increase was different among landraces. Specifically, the PE treatment of the two landraces with the higher yield, SM1 and SM3, increased this parameter of 21% and 35%, respectively. Overall, the added marketable yield (averaged across the four landraces) resulted from biostimulant applications was 6.64 t ha^−1^ compared to the control. Therefore, the positive effect associated with biostimulant treated plants resulted in an added gross return on San Marzano tomato value of 3320.0 € ha^−1^ (Figure 1). The total added variable cost associated with biostimulant applications was 1956.8 € ha^−1^ and was related particularly to hand harvesting of the added-tomato yield resulting in biostimulant-treated plants. After accounting for added variable costs, the net return of biostimulant-treated compared to untreated control plants was 1363.2 € ha^−1^ (Figure 1). As expected, the shape index was not altered by the growing conditions. Moreover, the PE significantly increased the citric acid in all the landraces but SM2, whose amount linearly correlated with a lower pH value of the fruit juice (Pearson Correlation: −0.492; *p* = 0.015). Acidity is an important factor for tomato flavor, although the limited difference in pH is not expected to considerably influence the suitability for tomato processing [34].

### 3.2. The Mineral Profile of Fruits Is Mainly Affected by Either the Genotype or Biostimulation

Inorganic ions represent a small fraction of the fruits’ dry matter. While the latter was unaffected by our experimental factors, mineral concentration in fruits was extensively and specifically altered by either the landraces or the biostimulation (Table 2). Two mineral elements (P and Ca) were not influenced by the two factors under investigation and their interaction. Nitrate, potassium, and magnesium concentrations varied according to the landrace and biostimulant application (Table 2). The SM3 landraces had a substantially higher content of these elements; for instance, almost double SM1 for nitrate. The effect of biostimulation was mineral-specific because it increased the fruit concentration of the two cations (Mg and K) and decreased that of the NO_3_ anion (Table 2). In addition to improving the nutritional value of the fruits (these elements being essential minerals for mammals), their higher accumulation indicates an enhanced mineral utilization efficiency. This is important especially for potassium, because of the possible reduction in the utilization rate of a chemical fertilizer that is required in higher quantity to produce whole peeled tomatoes [35]. While all these alterations were not influenced by the landrace factor, SM4 had a lower sulphate and sodium concentration compared to SM1, SM2 and SM3, irrespective of biostimulant treatment. Finally, mineral composition of the fruits was not shaped by the interaction of the genotype and the biostimulatory treatment. Overall, the data indicated that PE biostimulation can appreciably influence the nutritional value of tomato in a mineral-specific way. It was reported that an algal preparation specifically altered the mineral composition of cherry tomato fruits [36]. Our data indicate that for the different elements, either the landrace or PE biostimulation has a predominant effect. Relative variation in mineral composition was modest, except for nitrate. Anionic or cationic antagonisms were not evident, pointing towards a biostimulatory effect that should be also dependent on mineral transport to the fruits rather than exclusively influencing plant-soil interaction.

### 3.3. The Sugars and Bioactive Metabolites of the Fruits Are Specifically Changed by Biostimulation

Sugars are the predominant soluble solids of tomatoes and key contributors to their flavor [37]. The starch content in fruits was clearly different among varieties (CV: 23.8%). PE biostimulation had a clear positive effect (+73%) only for one landrace (SM3) while it does not significantly affect the other three (Table 3). Among the main free sugars, sucrose, and fructose, but not glucose, differed among landraces, with the SM3 having the highest content of these saccharides. The PE treatment had a positive effect on fructose and glucose (+13.6% and +26.9%, respectively), similarly to what has been reported in pepper, another Solanaceae [38]. The data indicated that the selected landraces have distinct accumulation patterns of sugars in mature fruits, specifically affected by biostimulation. Anthocyanins, and more generally polyphenols, did not vary according to the experimental factors. Differences among landraces were present in the lycopene concentration, and this variable was significantly increased (20.8%) by the biostimulation treatment. Even considering the limits of a comparison between different experimental works, this increase is higher than that observed with organic fertilizers [39]. In addition to the known beneficial effects for human health, the improvement of the lycopene content in mature fruits is an important trait for the processing industry, because of the resulting increase of the fruits’ red color intensity. Finally, for all these variables but starch, the effect of the biostimulant, when present, was not dependent on the landrace (Table 3).

### 3.4. Soluble Proteins and Free Amino Acids Profiling Were Principally Affected by the Landrace and Its Interaction with the Biostimulant

Both the variety and its interaction with the biostimulant significantly changed the soluble protein content. Specifically, the SM3 landraces had a significantly higher content of soluble proteins irrespective of the biostimulation. Moreover, in this landrace biostimulation strongly increased the soluble proteins content to a level that was higher than any other experimental conditions. Conversely, the treated SM1, SM2 and SM4 did not show a statistically significant difference from the control plants. Overall, PE biostimulation did not play a significant role in altering the nutritional value of the fruits in terms of total protein amount and free amino acids (AAs), including the essential AAs, except in SM3. This landrace turned out to be the most valuable genetic material along with SM2, which had the highest concentration of total AAs and essential AAs (among which BCAAs), while the lowest values were recorded for SM4. These parameters were not altered by the biostimulation (and factors’ interaction). The amount of the AAs was more dependent on the genotype factor (13 AAs significantly affected) than on the biostimulation (six AAs), and factors’ interaction (eight AAs). The effect of the PE biostimulation was overall positive, with four (respectively two) AAs present in higher (resp. lower) concentration. Compared to the other landraces, the SM1 had often a reduced quantity of free AAs and it is notable that proline was significantly higher, while other two other stress related AAs, GABA and monoethanolamine (MEA, had quantities similar to those of the other landraces. In the fruits of biostimulated SM1, total AAs content increased by about 50% compared to the respective control. Interestingly, biostimulation halved the amount of proline, while not affecting GABA.

Irrespective of the treatment, glutamate, glutamine, GABA, asparagine, and aspartate were the most abundant AAs, representing about 26.7%, 23.9%, 16.0%, 12.8% and 5.3% of total free AAs in all samples (Table 4). This profile is in agreement with that of the San Marzano varieties [40]. Therefore, it may be not casual that significant variations between the different SM landraces were observed for other AAs, such as arginine (SM2, SM3), MEA (SM1, SM2), lysine (SM2) and ornithine (SM2, SM4), which, along with GABA (SM1, SM2, SM3), were significantly higher in the indicated SM landraces compared to the other ones. Averaged over the different landraces, biostimulation significantly increased alanine and glycine concentration by 44.6% and 35.7%, respectively, compared to untreated plants. The highest asparagine content was present in SM3 that was equal to 8.34 ± 1.06 μmol g^−1^ FW. Biostimulant application increased the content of this AA only in SM1 (+2.7-fold compared to the untreated control). SM2 and SM3 had a glutamine content (14.7 μmol g^−1^ FW on average) higher than other two landraces. SM1 under control conditions had the highest proline of the four landraces, that was equal to 0.60 ± 0.08 μmol g^−1^ FW but decreased following biostimulation. SM2 had the highest content of essential AAs, which was on average 4.13 μmol g^−1^ FW. Only in the treated-SM1 fruits, essential AAs increased compared to the corresponding control, while no differences were observed in the other SME. The same trend was observed for the BCAAs content (Table 4).

### 3.5. Princpial Component Analysis Indicated the Complex Relationships of the Biostimulatory Response

**Figure 2:**
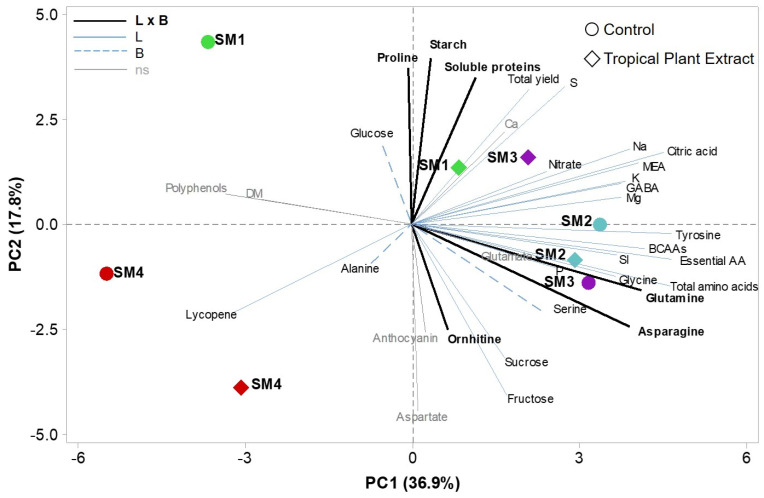
Principal Component Analysis bi plot. The first two PCs explained 54.7% of the total variance. Tropical plant extract-biostimulated landraces are represented by squares, control landraces by circles. Scaled loading vectors are drawn as black bold continuous line for variables that were signicantly affected by both the landrace and the biostimulant factors, blue continuous line for those significantly affected by the landraces, dashed blue line for those signifcanty affected by the biostimulant (only one occurrence) and as a thin gray line for variables that were not significantly influenced.

To highlight a possible underlying structure of the dataset, we summarized and visualized the various characteristics of the samples through multivariate analyses. Specifically, we used a Principal Component Analysis (PCA) to highlight patterns of variation from our range of different categories of measurements (Figure 2).

The PCA analysis indicated that the first four PCs had eigen values higher than one and explained 81.3% of the total variance, with PC1, PC2, PC3 and PC4 accounting for 36.9%, 17.8%, 16.0% and 10.5%, respectively. The first principal component had large positive associations with many AAs (e.g., tyrosine, essential AAs, BCAAs, amides and MEA), in addition to citric acid, and it was negatively correlated with lycopene (Figure 2). PC2 had a strong positive association with proline, starch, and soluble proteins content, while it was negatively correlated to fructose and sucrose content. Moreover, the loading plot indicated that proline content poorly correlated with the other AAs. The distribution of landraces based on the first two PCs indicated that trait variation was ample and able to disperse the samples along the two axes. Specifically, the SM1 and SM4 controls were present in distinct positions (i.e., SM1 in the upper and SM4 close to the negative side of PC1 in the lower left quadrant). The application of biostimulant strongly changed the distribution of these two landraces mainly along PC1 for SM1, and PC2 for SM4. Conversely, SM2 and SM3, present in the lower right quadrant close to *x*-axis, were primarily separated along the second axis, while the treated SM2 remained closer to its control and the treated SM3 moved in the upper right quadrant. Factor loadings and biplot analysis indicated that several, mostly uncorrelated, traits were the major determinants of observed diversity. The landrace scatter plot was not able to depict a clear pattern of grouping according to the genotype or the treatment. Overall, the multivariate analyses highlighted the biodiversity of tomato landraces (with similar geographical origin and destination of use) with respect to physicochemical, nutritional, and functional traits, which is evident also considering their different response to the PE biostimulant in non-stress conditions.

## 4. Conclusions

Our work provided evidence on the effect of a plant-based biostimulant on indeterminate, open-pollinated tomatoes that have been largely developed through adaptation rather than formal breeding. The targeted phytochemical profile indicated the presence of substantial variation among the landraces and their response to biostimulation, which proved to be capable of inducing landrace-specific beneficial features to yield and fruit quality. This observation would be consistent with a biostimulatory activity that is not acting towards one or few specific plant functions [41]. Under this perspective, our work showed that in tomato landraces, the influence of the tropical PE on the chemical and biochemical fruit composition is characterized by a substantial flexibility, in terms of both magnitude and type of altered parameters. Moreover, this response has a variable degree of correlation with the plant genotype, which may account for the sometimes-contrasting reports on biostimulation in tomato, and other species, under non-stress conditions [17,42]. The data also underlined our partial capacity to model fruit quality in response to biostimulation, an area that deserves further integrative investigations. Considering each single landrace, the scale of the modification (e.g., on dry weight, acidity, starch, free amino acids) implies that the tropical plant extract is not expected to affect largely the attributes of the berries that are important for their traditional destination of use (i.e., whole-peeled tomatoes). Interestingly, a positive impact of the biostimulation was present for some taste-related, nutritional, and functional quality traits of the fruits (e.g., simple sugars, lycopene, some organic acids and macroelements).

## Figures and Tables

**Table 1:** Yield, shape index, dry matter percentage, pH and citrate content of the fruits in relation to the landraces (SM1, SM2, SM3, SM4) and the biostimulation application. All data are expressed as mean ± SE, *n* = 3.

Source of Variance	Yield		Shape Index		Dry Matter		pH		Citrate	
	(t ha^−1^)				(%)				(g kg^−1^ DW)	
Landrace (L)										
SM1	46.68	±2.73 a	1.68	±0.09 b	5.71	±0.09	4.31	±0.02 bc	64.91	±3.30 b
SM2	36.62	±2.10 ab	2.09	±0.07 a	5.66	±0.11	4.35	±0.03 ab	73.57	±2.60 a
SM3	47.62	±4.23 a	2.20	±0.11 a	5.34	±0.22	4.23	±0.04 c	74.11	±3.37 a
SM4	31.10	±3.14 b	1.79	±0.12 b	5.83	±0.10	4.42	±0.03 a	47.24	±1.21 c
Biostimulant (B)										
Control	37.18	±2.30	1.94	±0.23	5.53	±0.12	4.34	±0.03	60.97	±3.60
Plant Extract	43.82	±2.06	1.95	±0.26	5.74	±0.08	4.32	±0.02	68.95	±3.54
Significance										
Landrace (L)	**		***		ns		**		***	
Biostimulant (B)	*		ns		ns		ns		**	
L × B	ns		ns		ns		ns		ns	

ns, *, **, *** Nonsignificant or significant at *p* ≤ 0.05, 0.01, and 0.001, respectively. Different letters within each column indicate significant differences according to Duncan’s multiple-range test (*p* < 0.05). The factor “Biostimulant” was compared with the Student’s *t*-test.

**Table 2:** Mineral composition of the tomato fruits in relation to the landraces (SM1, SM2, SM3, SM4) and the biostimulation application. All data are expressed as mean ± SE, *n* = 3.

Source of Variance	NO_3_^−^N		P		K		Ca		Mg		S		Na	
	(mg kg^−1^ FW)		(g kg^−1^ DW)		(g kg^−1^ DW)		(g kg^−1^ DW)		(g kg^−1^ DW)		(g kg^−1^ DW)		(g kg^−1^ DW)	
Landrace (L)														
SM1	77.71	±5.78 ab	3.20	±0.18	49.21	±2.10 ab	0.96	±0.05	1.97	±0.05 b	0.84	±0.04 a	0.26	±0.02 a
SM2	67.75	±3.85 bc	3.26	±0.20	53.55	±1.32 ab	0.96	±0.05	2.30	±0.10 a	0.83	±0.03 a	0.28	±0.02 a
SM3	91.05	±9.92 a	3.18	±0.23	57.93	±5.38 a	0.85	±0.05	2.34	±0.15 a	0.77	±0.06 a	0.24	±0.02 a
SM4	51.74	±6.03 c	3.03	±0.21	44.47	±0.69 b	0.81	±0.06	1.89	±0.10 b	0.64	±0.04 b	0.17	±0.01 b
Biostimulant (B)														
Control	80.04	±6.92	3.21	±0.16	48.10	±1.31	0.93	±0.04	2.02	±0.08	0.73	±0.04	0.24	±0.02
Tropical plant extract	64.08	±4.42	3.13	±0.12	54.48	±3.01	0.86	±0.04	2.22	±0.09	0.80	±0.03	0.23	±0.01
Significance														
Landrace (L)	**		ns		*		ns		**		**		***	
Biostimulant (B)	*		ns		*		ns		*		ns		ns	
L × B	ns		ns		ns		ns		ns		ns		ns	

ns, *, **, *** Nonsignificant or significant at *p* ≤ 0.05, 0.01, and 0.001, respectively. Different letters within each column indicate significant differences according to Duncan’s multiple-range test (*p* < 0.05). The factor “Biostimulant” was compared with the Student’s *t*-test.

**Table 3:** Starch, free sugars, anthocyanins, lycopene, and polyphenols in relation to the landraces (SM1, SM2, SM3, SM4) and the biostimulation application. All data are expressed as mean ± SE, *n* = 3.

Source of Variance	Starch		Glucose		Fructose		Sucrose		Anthocyanins		Lycopene		Polyphenols	
	(µmol g^−1^ FW)		(µmol g^−1^ FW)		(µmol g^−1^ FW)		(µmol g^−1^ FW)		(mg C3G 100 g^−1^ FW)		(mg 100 g^−1^ FW)		(mg GAE 100 g^−1^ FW)	
Landrace (L)														
SM1	31.99	±2.58 a	64.49	±1.56	14.18	±2.75 b	21.73	±2.49 b	44.60	±2.12	15.73	±2.15 ab	37.03	±1.98
SM2	20.48	±1.04 c	58.27	±3.54	28.42	±2.71 a	23.90	±2.04 b	50.44	±2.87	13.47	±1.22 b	34.97	±2.35
SM3	26.25	±3.84 b	63.10	±5.74	26.61	±2.22 a	29.87	±2.31 a	47.50	±2.06	11.78	±1.18 b	36.10	±3.47
SM4	19.28	±0.25 c	60.88	±2.45	27.76	±1.23 a	27.12	±1.21 ab	49.93	±3.90	19.53	±1.83 a	38.70	±2.15
Biostimulant (B)														
Control	22.68	±1.34	57.76	±2.60 b	21.37	±2.60	23.92	±2.03	47.56	±1.44	13.70	±1.27	36.20	±1.76
Tropical plant extract	26.32	±2.72	65.61	±1.92 a	27.12	±1.68	27.39	±1.00	48.68	±2.48	16.55	±1.42	37.21	±1.78
Significance														
Landrace (L)	***		ns		***		*		ns		**		ns	
Biostimulant (B)	ns		*		*		ns		ns		*		ns	
L × B	*		ns		ns		ns		ns		ns		ns	

ns, *, **, *** Nonsignificant or significant at *p* ≤ 0.05, 0.01, and 0.001, respectively. Different letters within each column indicate significant differences according to Duncan’s multiple-range test (*p* < 0.05). The factor “Biostimulant” was compared with the Student’s *t*-test.

**Table 4:** Amino acidic profile of the fruits in relation to the landraces (SM1, SM2, SM3, SM4) and the biostimulation applications. All data are expressed as mean ± standard error, *n* = 3.

Chemical Compound	Landrace					Biostimulant			Landrace × Biostimulant								
	SM1	SM2	SM3	SM4	sig	Control (C)	Biostimulation (PE)	sig	SM1 C	SM2 C	SM3 C	SM4 C	SM1 PE	SM2 PE	SM3 PE	SM4 PE	sig
Soluble proteins ^a^	26.15 ± 2.29 ab	22.75 ± 1.49 bc	30.05 ± 3.48 a	20.05 ± 1.82 c	**	24.49 ± 1.32	25.01 ± 2.45	ns	27.94 ± 3.63 b	25.66 ± 1.36 bc	22.90 ± 0.38 bc	21.45 ± 3.26 bc	24.36 ± 3.15 bc	19.83 ± 0.87 bc	37.20 ± 3.04 a	18.65 ± 1.98 c	**
Alanine ^b^	1.22 ± 0.13	1.07 ± 0.15	0.68 ± 0.07	1.11 ± 0.26	ns	0.83 ± 0.11	1.20 ± 0.12	*	1.14 ± 0.26	0.95 ± 0.26	0.65 ± 0.10	0.59 ± 0.15	1.29 ± 0.11	1.18 ± 0.16	0.71 ± 0.13	1.63 ± 0.20	ns
Arginine ^b^	0.85 ± 0.05 b	1.55 ± 0.15 a	1.35 ± 0.20 a	0.55 ± 0.06 b	***	1.06 ± 0.16	1.08 ± 0.13	ns	0.74 ± 0.03	1.56 ± 0.08	1.49 ± 0.32	0.46 ± 0.11	0.95 ± 0.04	1.54 ± 0.31	1.21 ± 0.27	0.63 ± 0.02	ns
Asparagine ^b^	4.41 ± 0.92 c	6.76 ± 0.50 ab	8.34 ± 1.06 a	5.18 ± 0.72 bc	**	5.83 ± 0.95	6.52 ± 0.35	ns	2.43 ± 0.41 c	7.02 ± 0.93 ab	9.52 ± 1.84 a	4.35 ± 0.92 bc	6.39 ± 0.34 ab	6.49 ± 0.55 ab	7.17 ± 0.91 ab	6.02 ± 1.04 b	*
Aspartate ^b^	2.32 ± 0.27	2.44 ± 0.20	2.59 ± 0.33	2.87 ± 0.38	ns	2.37 ± 0.24	2.74 ± 0.17	ns	1.90 ± 0.13	2.17 ± 0.25	2.85 ± 0.54	2.56 ± 0.76	2.75 ± 0.42	2.70 ± 0.26	2.33 ± 0.44	3.17 ± 0.22	ns
MEA ^b^	0.10 ± 0.01 a	0.12 ± 0.01 a	0.09 ± 0.01 ab	0.07 ± 0.01 b	*	0.09 ± 0.01	0.09 ± 0.01	ns	0.09 ± 0.01	0.11 ± 0.01	0.10 ± 0.01	0.07 ± 0.01	0.11 ± 0.02	0.12 ± 0.03	0.09 ± 0.01	0.06 ± 0.01	ns
GABA ^b^	7.89 ± 0.92 a	10.33 ± 1.43 a	7.95 ± 0.91 a	4.84 ± 0.50 b	**	7.21 ± 0.67	8.29 ± 1.04	ns	7.05 ± 1.53	8.33 ± 0.62	8.55 ± 1.51	4.92 ± 0.90	8.73 ± 1.08	12.33 ± 2.42	7.34 ± 1.22	4.75 ± 0.67	ns
Glycine ^b^	0.12 ± 0.01 b	0.20 ± 0.02 a	0.20 ± 0.03 a	0.14 ± 0.02 b	**	0.14 ± 0.02	0.19 ± 0.02	*	0.10 ± 0.01	0.21 ± 0.02	0.16 ± 0.02	0.10 ± 0.02	0.14 ± 0.01	0.20 ± 0.03	0.24 ± 0.05	0.18 ± 0.03	ns
Glutamate ^b^	13.75 ± 1.79	13.66 ± 1.87	12.45 ± 1.89	11.81 ± 1.40	ns	12.01 ± 1.03	13.82 ± 1.29	ns	11.42 ± 1.21	10.57 ± 0.45	14.24 ± 3.19	11.81 ± 2.83	16.09 ± 3.01	16.74 ± 2.78	10.66 ± 2.11	11.81 ± 1.33	ns
Glutamine ^b^	7.94 ± 1.21 b	13.76 ± 1.79 a	15.64 ± 1.18 a	8.88 ± 1.33 b	***	11.51 ± 1.71	11.60 ± 0.87	ns	5.77 ± 0.50 d	16.72 ± 1.84 a	16.83 ± 1.53 a	6.74 ± 1.64 cd	10.12 ± 1.55 bcd	10.80 ± 1.96 bcd	14.45 ± 1.80 ab	11.02 ± 1.25 bc	*
Isoleucine ^b^	0.43 ± 0.05	0.57 ± 0.06	0.42 ± 0.04	0.36 ± 0.04	ns	0.41 ± 0.04	0.49 ± 0.04	ns	0.34 ± 0.04	0.53 ± 0.10	0.42 ± 0.05	0.33 ± 0.08	0.53 ± 0.06	0.60 ± 0.09	0.43 ± 0.07	0.39 ± 0.00	ns
Histidine ^b^	0.43 ± 0.06 bc	0.61 ± 0.04 a	0.53 ± 0.07 ab	0.35 ± 0.04 c	*	0.47 ± 0.05	0.49 ± 0.04	ns	0.35 ± 0.06	0.61 ± 0.03	0.62 ± 0.11	0.31 ± 0.07	0.51 ± 0.08	0.61 ± 0.09	0.44 ± 0.05	0.38 ± 0.04	ns
Leucine ^b^	0.37 ± 0.04 b	0.48 ± 0.02 a	0.33 ± 0.02 b	0.32 ± 0.03 b	**	0.35 ± 0.03	0.40 ± 0.03	ns	0.30 ± 0.03	0.47 ± 0.02	0.36 ± 0.02	0.27 ± 0.04	0.43 ± 0.07	0.49 ± 0.03	0.31 ± 0.04	0.36 ± 0.04	ns
Lysine ^b^	0.40 ± 0.06 b	0.60 ± 0.05 a	0.43 ± 0.04 b	0.35 ± 0.05 b	**	0.44 ± 0.05	0.46 ± 0.03	ns	0.29 ± 0.04 d	0.67 ± 0.06 a	0.49 ± 0.08 abc	0.31 ± 0.06 cd	0.52 ± 0.04 ab	0.54 ± 0.05 ab	0.38 ± 0.02 bcd	0.39 ± 0.09 bcd	*
Methionine ^b^	0.07 ± 0.01 bc	0.11 ± 0.00 a	0.08 ± 0.01 b	0.05 ± 0.01 c	***	0.07 ± 0.01	0.09 ± 0.01	*	0.05 ± 0.00 de	0.12 ± 0.01 a	0.08 ± 0.01 cd	0.04 ± 0.01 e	0.09 ± 0.01 bc	0.11 ± 0.00 ab	0.08 ± 0.01 cd	0.07 ± 0.01 cd	*
Ornhitine ^b^	0.20 ± 0.03 b	0.32 ± 0.06 a	0.20 ± 0.02 b	0.30 ± 0.03 a	**	0.27 ± 0.03	0.25 ± 0.02	ns	0.16 ± 0.00 c	0.44 ± 0.06 a	0.21 ± 0.02 c	0.25 ± 0.01 bc	0.24 ± 0.04 c	0.20 ± 0.03 c	0.20 ± 0.02 c	0.35 ± 0.04 ab	***
Phenylalanine ^b^	0.68 ± 0.09	0.85 ± 0.09	0.66 ± 0.09	0.68 ± 0.11	ns	0.63 ± 0.05	0.80 ± 0.07	ns	0.54 ± 0.08	0.73 ± 0.04	0.69 ± 0.15	0.55 ± 0.13	0.81 ± 0.11	0.97 ± 0.15	0.62 ± 0.13	0.81 ± 0.17	ns
Proline ^b^	0.40 ± 0.10 a	0.30 ± 0.03 b	0.20 ± 0.03 c	0.11 ± 0.00 c	***	0.31 ± 0.06	0.19 ± 0.03	**	0.60 ± 0.08 a	0.28 ± 0.05 bc	0.25 ± 0.02 bcd	0.12 ± 0.00 de	0.20 ± 0.02 bcde	0.32 ± 0.05 b	0.15 ± 0.03 cde	0.10 ± 0.01 e	***
Serine ^b^	0.90 ± 0.10	1.18 ± 0.04	1.06 ± 0.16	0.99 ± 0.27	ns	0.82 ± 0.09	1.25 ± 0.10	**	0.72 ± 0.11	1.16 ± 0.01	0.91 ± 0.16	0.49 ± 0.12	1.08 ± 0.07	1.20 ± 0.10	1.21 ± 0.28	1.49 ± 0.29	ns
Tyrosine ^b^	0.22 ± 0.02 b	0.38 ± 0.03 a	0.34 ± 0.02 a	0.18 ± 0.02 b	***	0.26 ± 0.03	0.30 ± 0.03	ns	0.19 ± 0.03	0.36 ± 0.06	0.34 ± 0.03	0.16 ± 0.03	0.25 ± 0.02	0.40 ± 0.04	0.34 ± 0.05	0.20 ± 0.04	ns
Threonine ^b^	0.28 ± 0.04	0.34 ± 0.08	0.38 ± 0.08	0.23 ± 0.02	ns	0.29 ± 0.05	0.33 ± 0.04	ns	0.21 ± 0.03	0.28 ± 0.10	0.43 ± 0.14	0.22 ± 0.04	0.35 ± 0.06	0.40 ± 0.14	0.32 ± 0.11	0.24 ± 0.03	ns
Tryptophan ^b^	0.09 ± 0.01 c	0.16 ± 0.01 ab	0.17 ± 0.03 a	0.13 ± 0.01 b	***	0.15 ± 0.02	0.13 ± 0.01	*	0.08 ± 0.00 d	0.17 ± 0.02 b	0.23 ± 0.01 a	0.14 ± 0.02 bc	0.11 ± 0.01 cd	0.15 ± 0.01 bc	0.12 ± 0.01 c	0.13 ± 0.01 bc	***
Valine ^b^	0.23 ± 0.04 c	0.41 ± 0.03 a	0.31 ± 0.02 b	0.18 ± 0.02 c	***	0.27 ± 0.04	0.30 ± 0.02	ns	0.17 ± 0.02 d	0.45 ± 0.03 a	0.30 ± 0.02 bc	0.15 ± 0.02 d	0.30 ± 0.04 bc	0.37 ± 0.03 ab	0.32 ± 0.05 b	0.22 ± 0.00 cd	*
Essential AA ^b^	3.84 ± 0.41 bc	5.68 ± 0.36 a	4.67 ± 0.49 ab	3.21 ± 0.30 c	**	4.14 ± 0.42	4.56 ± 0.34	ns	3.07 ± 0.25	5.58 ± 0.23	5.11 ± 0.73	2.80 ± 0.43	4.61 ± 0.44	5.78 ± 0.77	4.23 ± 0.70	3.62 ± 0.30	ns
BCAAs ^b^	1.03 ± 0.13 b	1.45 ± 0.07 a	1.07 ± 0.08 b	0.86 ± 0.08 b	**	1.02 ± 0.09	1.19 ± 0.08	ns	0.81 ± 0.08	1.44 ± 0.11	1.08 ± 0.09	0.76 ± 0.13	1.26 ± 0.17	1.46 ± 0.12	1.06 ± 0.15	0.97 ± 0.03	ns
Total amino acids ^b^	43.31 ± 4.94 ab	56.20 ± 4.10 a	54.42 ± 5.36 a	39.67 ± 4.42 b	*	45.80 ± 4.31	51.00 ± 3.10	ns	34.63 ± 4.05	53.92 ± 2.64	59.71 ± 8.90	34.94 ± 7.43	51.99 ± 5.51	58.48 ± 8.47	49.13 ± 6.05	44.40 ± 4.49	ns

^a^: mg g^−1^ FW; ^b^: µmol g^−1^ FW; ns, *, **, *** Nonsignificant or significant at *p* ≤ 0.05, 0.01, and 0.001, respectively. Different letters within each row indicate significant differences according to Duncan’s multiple-range test (*p* < 0.05). The factor “Biostimulant” was compared with the Student’s *t*-test.

## Data Availability

The datasets generated for this study are available on request to the corresponding author.

## Supplementary Materials

The following are available online at https://www.mdpi.com/article/10.3390/foods10050926/s1, Supplementary Table S1. Morphological characteristics of the four SM landraces. Quantitative traits are reported as mean ± standard error, *n* = 3.
