# Supplementary material for: Biostimulation as a Means for Optimizing Fruit Phytochemical Content and Functional Quality of Tomato Landraces of the San Marzano Area
Source: Foods. 2021 Apr 23;10(5):926. doi: 10.3390/foods10050926 (PMC8145630; doi:10.3390/foods10050926)
Supplement: Supplementary file 1 [file foods-10-00926-s001.zip › foods-1164402-supplementary.pdf]

foods

MDPI

Supplementary Table S1. Morphological characteristics of the four SM landraces. Quantitative traits are reported as mean ± standard error, n = 3.

Landrace	Site of collection	Plant growth type	Fruit in longitudinal section			Fruit shape at blossom end	Longitudinal length (cm)	Equatorial length (cm)
			Broadest part	Shape	Length/width			
SM1	Bellizzi (SA)	indeterminate	below middle	ovate	narrow	flat	6.61 ± 0.05	3.95 ± 0.10
SM2	Bellizzi (SA)	indeterminate	at middle	elliptic	narrow	pointed	7.32 ± 0.14	3.51 ± 0.07
SM3	Sarno (SA)	indeterminate	at middle	cylindric	narrow	pointed	8.13 ± 0.26	3.69 ± 0.08
SM4	Montoro Inferiore (AV)	indeterminate	at middle	cylindric	narrow	flat to pointed	7.44 ± 0.22	4.16 ± 0.11
